# Delayed Chemotherapy-Induced Nausea and Vomiting: Pathogenesis, Incidence, and Current Management

**DOI:** 10.3389/fphar.2017.00019

**Published:** 2017-01-30

**Authors:** Bernardo L. Rapoport

**Affiliations:** The Medical Oncology Centre of RosebankJohannesburg, South Africa

**Keywords:** antiemetics, delayed chemotherapy-induced nausea and vomiting, emesis, highly emetogenic chemotherapy, moderately emetogenic chemotherapy, nausea, neurokinin-1 receptor antagonists, vomiting

## Abstract

Even when chemotherapy-induced nausea and vomiting (CINV) can be effectively controlled in the acute phase, it may still occur in the delayed phase. Identifying at-risk patients is complex and requires consideration of clinical, personal, demographic, and behavioral factors. Delayed CINV has a significant detrimental effect on patients’ daily life and is responsible for significant healthcare resource utilization. Patients who do not experience acute CINV are not necessarily exempt from delayed CINV, and healthcare professionals have been shown to underestimate the incidence of delayed CINV. Failure to protect against CINV during the first cycle of chemotherapy is the most significant independent risk factor for delayed CINV during subsequent cycles. Addition of a neurokinin-1 receptor antagonist to antiemetic prophylactic regimens involving a 5-hydroxytryptamine type 3 receptor antagonist and a corticosteroid helps to ameliorate delayed CINV, particularly vomiting. Netupitant and rolapitant are second-generation neurokinin-1 receptor antagonists that provide effective prophylaxis against delayed chemotherapy-induced vomiting and also have an antinausea benefit. All of the neurokinin-1 receptor antagonists with the exception of rolapitant inhibit or induce cytochrome P450 3A4 (CYP3A4), and a reduced dose of dexamethasone (a CYP3A4 substrate) should be administered with aprepitant or netupitant; by contrast, this is not necessary with rolapitant. Here we review specific challenges associated with delayed CINV, its pathophysiology, epidemiology, treatment, and outcomes relative to acute CINV, and its management within the larger context of overall CINV.

## Introduction

Nausea and vomiting are the most feared side effects of cytotoxic chemotherapy (de Boer-Dennert et al., 1997; Sun et al., 2005) and can have a deleterious effect on health-related quality of life (Bloechl-Daum et al., 2006; Hilarius et al., 2012), compromise treatment outcomes (Vidall et al., 2011; Jordan et al., 2015; Van Laar et al., 2015; National Comprehensive Cancer Network, 2016; Navari, 2016), and increase healthcare resource utilization (Schwartzberg L. et al., 2015). Chemotherapy-induced nausea and vomiting (CINV) typically presents in two phases, the acute phase and the delayed phase, over a 5-day period (Navari and Aapro, 2016). Acute CINV occurs within 1–2 h of chemotherapy administration and can last for up to 24 h; delayed CINV presents more than 24 h after chemotherapy administration, and it is most frequently reported with the agents cisplatin, carboplatin, cyclophosphamide, and doxorubicin (National Comprehensive Cancer Network, 2016).

While acute CINV is reasonably well managed with serotonin (5-hydroxytryptamine) type 3 (5-HT_3_) receptor antagonists in the majority of patients (Jordan et al., 2007), delayed CINV continues to present a treatment challenge (Grunberg et al., 2004; Hsieh et al., 2015; Baba et al., 2016). This review discusses the pathophysiology, burden of illness, and treatment outcomes associated with delayed CINV, together with advances and future directions for management.

### The Pathophysiology of Delayed CINV

Chemotherapy-induced nausea and vomiting is a highly complex reflex that involves contributory pathways from both the central and peripheral nervous systems. While the pathophysiology of emesis is not completely understood, it is currently thought that chemotherapy-induced release of neurotransmitters stimulates receptors on the terminals of afferent nerves in various locations, including the gastrointestinal tract, cerebral cortex and thalamus, vestibular region, and area postrema, which project to the nucleus tractus solitarius (NTS) located in the brain stem (Aapro et al., 2014a; Babic and Browning, 2014; National Comprehensive Cancer Network, 2016; Navari and Aapro, 2016). The NTS plays a dominant role in coordinating the autonomic processes involved in vomiting, such as swallowing, salivation, respiration, abdominal muscle contraction and relaxation, and intestinal contraction and relaxation (Babic and Browning, 2014). In addition, neurotransmitters may directly stimulate receptors located in the area postrema of the brain (known as the chemoreceptor trigger zone), which also activates the NTS (Aapro et al., 2014a). Neurotransmitters that have been identified as important mediators of CINV include serotonin and substance P (Navari and Aapro, 2016).

**FIGURE 1:**
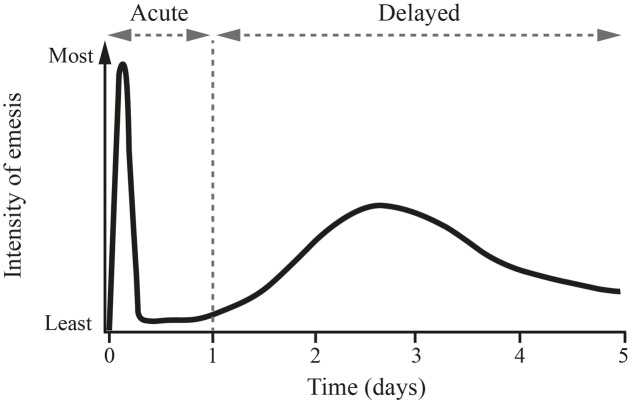
**Pattern of cisplatin-induced delayed emesis**. This illustrates the biphasic pattern of emesis after the administration of high-dose cisplatin, with the maximum intensity seen with the initial 24 h, followed by a second peak of less intense nausea and emesis on days 2 and 3. Reprinted from *Springer Drugs* 1996 Nov; 52 (5): 639–648, Tavorath R and Hesketh PJ (Adis International Limited. All rights reserved). With permission of Springer.

The typical pattern of CINV is shown in **Figure 1**. The acute phase occurs within the first 24 h after chemotherapy and is largely mediated by 5-HT_3_ receptors in the intestine (Navari, 2016). In this phase, free radicals generated after administration of chemotherapy induce the release of serotonin from enterochromaffin cells located in the intestinal mucosa (Aapro et al., 2014a; Navari, 2016). Serotonin then interacts with 5-HT_3_ receptors located on vagal afferent nerves in the intestinal wall, which project to the area postrema and NTS, stimulating the vomiting reflex (Aapro et al., 2014a). Serotonin may also directly interact with 5-HT_3_ receptors on the area postrema (Aapro et al., 2014a). Acute CINV is therefore particularly sensitive to 5-HT_3_ receptor antagonists (Aapro, 2005) (**Figure 1**); however, these agents have little impact on delayed CINV (Aapro, 2005), suggesting that different pathophysiologic mechanisms may be at play during the second emetic phase.

The delayed phase of CINV starts on day 2 after chemotherapy and can last up to day 5. Delayed CINV is predominantly driven by a central pathway involving the neurotransmitter/neuromodulator substance P, which is a member of the mammalian tachykinin family of peptides (Garcia-Recio and Gascon, 2015). Substance P is released from neurons in response to chemotherapy and binds to neurokinin-1 (NK-1) receptors in the area postrema and NTS, thereby mediating the induction of vomiting (Armstrong et al., 1981; Aapro et al., 2014a). The dominant role of substance P in delayed CINV is demonstrated by the effectiveness of NK-1 receptor antagonists in preventing CINV during this phase (Navari, 2016) (**Figure 1**). NK-1 receptors are also located on vagal afferent terminals in the gastrointestinal tract, suggesting that substance P released from enterochromaffin cells in response to chemotherapy may also play an auxiliary role in the acute phase of CINV (Hesketh, 2008).

### Classification of Emetic Agents

The emetogenicity of chemotherapy refers to its capacity to induce nausea and vomiting when administered without adequate antiemetic prophylaxis. One of the most commonly used schemes divides chemotherapeutic agents into four categories (high, moderate, low, and minimal), depending on the percentage of patients who would experience emesis in the acute phase while receiving the agent without adequate antiemetic prophylaxis (Hesketh et al., 1997). In the absence of such prophylaxis, it is estimated that over 90% of patients exposed to highly emetogenic chemotherapy (HEC) and between 30 and 90% of patients exposed to moderately emetogenic chemotherapy (MEC) will experience acute-phase CINV (**Table 1**). Emetogenic categories are regularly updated by guidelines groups to incorporate new agents or new data from existing agents (Basch et al., 2011; Hesketh et al., 2016a; National Comprehensive Cancer Network, 2016). One particularly significant change for CINV was the reclassification of anthracycline-cyclophosphamide (AC)–based chemotherapy from the moderately emetogenic category to the highly emetogenic category in 2011. In addition, while carboplatin-based chemotherapy is defined as MEC, the Multinational Association of Supportive Care in Cancer (MASCC) and European Society for Medical Oncology (ESMO) guidelines were recently updated to recommend that CINV associated with carboplatin therapy be treated in the same way as HEC, with an NK-1 receptor antagonist as well as a 5-HT_3_ receptor antagonist and dexamethasone (MASCC/ESMO, 2016).

**Table 1:** Emetogenic risk categories for antineoplastic agents (based on acute emetogenicity) (Hesketh et al., 1997).

Emetogenic risk	Frequency of emesis in the absence of effective antiemetic prophylaxis	Intravenous antineoplastic agents
**High**	>90%	• AC combination: doxorubicin or epirubicin + cyclophosphamide
		• Carmustine >250 mg/m^2^
		• Cisplatin^2^
		• Cyclophosphamide >1,500 mg/m^2^
		• Dacarbazine
		• Doxorubicin ≥60 mg/m^2^
		• Epirubicin ≥90 mg/m^2^
		• Ifosfamide ≥2 g/m^2^ per dose
		• Mechlorethamine
		• Streptozocin
**Moderate**	30–90%	• Aldesleukin >12–15 million IU/m^2^
		• Amifostine >300 mg/m^2^
		• Arsenic trioxide
		• Bendamustine
		• Busulfan
		• Carboplatin^a^
		• Carmustine^a^ ≤250 mg/m^2^
		• Cloforabine
		• Cyclophosphamide <1,500 mg/m^2^
		• Cytarabine >200 mg/m^2^
		• Dactinomycin^a^
		• Daunorubicin^a^
		• Dinutuximab
		• Doxorubicin^a^ <60 mg/m^2^
		• Epirubicin^a^ <90 mg/m^2^
		• Idarubicin
		• Ifosfamide^a^ <2 g/m^2^ per dose
		• Interferon-alfa ≥10 million IU/m^2^
		• Irinotecan^a^
		• Melphalan
		• Methotrexate^a^ ≥250 mg/m^2^
		• Oxaliplatin
		• Temozolomide
		• Trabectedin

^a^*May be highly emetogenic in some patients*.

It should be noted that emetogenic categories are based only on the incidence of acute CINV, rather than delayed or overall CINV. Indeed, one recent study found that the chemotherapy regimen is an inconsistent predictor of CINV in the delayed phase (Jordan et al., 2014). This suggests that emetogenic classifications may not be the most appropriate determinant of prophylactic antiemetic regimen for delayed CINV and may contribute to undertreatment of delayed CINV due to a lack of appreciation of the true emetogenic risk of particular types of chemotherapy.

### Epidemiology and Risk Factors for Delayed CINV

Chemotherapy-induced nausea and vomiting may be more problematic during the delayed phase than the acute phase in patients receiving HEC and MEC (Grunberg et al., 2004; Escobar et al., 2015; Hsieh et al., 2015; Baba et al., 2016). For example, in an international, prospective observational study of 298 adult patients receiving chemotherapy for the first time, delayed nausea and vomiting were observed in 60 and 50% of HEC patients, respectively, and in 52 and 28% of MEC patients, respectively (Grunberg et al., 2004), whereas acute nausea and vomiting were seen in 12 and 33% of HEC patients, respectively, and in 13 and 37% of MEC patients, respectively. The majority of patients were receiving antiemetic prophylaxis according to then-current guidelines, with 97% receiving a 5-HT_3_ receptor antagonist and 78% a corticosteroid. The study concluded that patients who do not experience acute CINV are not necessarily protected from delayed CINV: 24 and 23% of patients reported delayed nausea and emesis, respectively, even in the absence of these events in the acute phase. Similar patterns were observed among patients assigned to HEC and MEC. In another study in 240 chemotherapy-naive patients in Spain who received MEC with a 5-HT_3_ receptor antagonist plus corticosteroid antiemetic prophylaxis, the incidence of CINV was higher in the delayed phase than in the acute phase (Escobar et al., 2015). This difference was statistically significant for the endpoints of vomiting, nausea, and significant nausea, with rates increasing from 9.2 to 16.5% (*p* = 0.0112), from 23.3 to 38.5% (*p* < 0.0001), and from 9.4 to 21.7% (*p* = 0.0002), respectively. Twice as many patients required rescue antiemetics (metoclopramide or ondansetron) during the delayed phase (14.5%) as during the acute phase (7.2%).

More recent observational studies continue to show that delayed CINV may be incompletely controlled even if acute CINV is adequately managed (Molassiotis et al., 2014; Hsieh et al., 2015). In a large, heterogeneous group of European cancer patients (*n* = 991) receiving their first cycle of routine HEC or MEC, complete response (CR) rates (the proportion of patients without vomiting or significant nausea) were 72% in the acute phase and 62% in the delayed phase (Jordan et al., 2014). The delayed-phase CR rate increased to 67% by cycle 3 (*p* = 0.0144 compared with cycle 1); this improvement seemed to be driven by patients reporting no vomiting (71% in cycle 1 and 78% in cycle 3; *p* < 0.0001) rather than those reporting no significant nausea (81% in cycle 1 and 81% in cycle 3).

Survey data indicates that oncologists and oncology nurses can accurately predict the incidence of acute CINV after HEC; however, the incidence of delayed CINV after HEC is often underestimated. In one study, the predicted incidence of delayed nausea was 39% (95% confidence interval [CI] 30–48%), whereas the observed incidence was 60% (95% CI 48–72%), and the predicted incidence of delayed vomiting was 22% (95% CI 12–31%), whereas the observed incidence was 50% (95% CI 37–63%) (Grunberg et al., 2004). Delayed CINV has also been underestimated in patients receiving MEC (Grunberg et al., 2004; Escobar et al., 2015). Misperceptions regarding the incidence of delayed CINV may have implications for treatment. For example, in a hospital-based study in Spain, patients who experienced acute vomiting in cycle 1 were significantly more likely to have a change in antiemetic therapy in subsequent cycles; by contrast, delayed vomiting or nausea at any stage did not lead to changes in subsequent antiemetic regimens (Molassiotis et al., 2008).

Identifying which patients are at greatest risk for CINV is a complex analysis combining clinical, personal, demographic, and behavioral characteristics. A number of factors have been identified that increase susceptibility to CINV, including female sex, age <55 years, a history of nausea/vomiting, anxiety, fatigue or motion sickness, impaired quality of life, and limited alcohol use (Dranitsaris et al., 2013; Jordan et al., 2014). One study evaluated independent risk factors for the development of delayed CINV during cycle 1 of chemotherapy, identifying guideline-inconsistent CINV prophylaxis, no use of secondary antiemetics for delayed CINV, a history of nausea/vomiting, and prechemotherapy (anticipatory) nausea (Jordan et al., 2014). Other factors that have been associated with delayed CINV include a history of motion sickness, acute CINV, and the use of cisplatin (Kottschade et al., 2016). A small prospective study of 56 cancer patients (Higgins et al., 2007) also noted a significant relationship between pretreatment distress and the severity of subsequent delayed nausea but not acute nausea.

Studies evaluating multiple cycles of chemotherapy have revealed that an important predictor of CINV in a given cycle is whether CINV occurred in a previous cycle. A study of patients from Italian oncology centers receiving ondansetron or metoclopramide for cisplatin-associated CINV found that protection from emesis during the first cycle of cisplatin-based chemotherapy was an important predictor of protection in subsequent cycles (Italian Group for Antiemetic Research, 1994). Jordan et al. (2014) showed that the most significant independent risk factor for delayed CINV during cycles 2 and 3 was not achieving a CR in the previous cycle: patients without a CR in the earlier cycle were 5.7–7.3 times more likely to have no CR during the delayed phase in the subsequent cycle. Furthermore, the failure to protect against delayed CINV in the first cycle of chemotherapy can impair protection against acute CINV in subsequent cycles (Ellebaek and Herrstedt, 2008). These findings underscore the importance of effective management of delayed CINV during the first cycle of chemotherapy.

### Clinical Implications of Delayed CINV

Delayed CINV has a significant detrimental effect on a patient’s daily life (Bloechl-Daum et al., 2006; Hilarius et al., 2012; Grassi et al., 2015), even in the absence of acute CINV. In a representative sample of 298 treatment-naive patients receiving HEC or MEC and given CINV prophylaxis under then-current patterns of clinical practice, the impact of CINV on daily life was assessed using the Functional Living Index–Emesis questionnaire on day 6 of cycle 1 (Bloechl-Daum et al., 2006). Only 32% of patients who experienced delayed vomiting without acute vomiting reported that CINV had no or minimal impact on daily life, similar to the proportion of patients who experienced only acute vomiting (30%). In the same study, 80% of patients who experienced acute nausea without delayed nausea reported that emesis did not affect their daily life; by contrast, only 56% of those who experienced delayed nausea without acute nausea reported no or minimal impact.

Delayed CINV is also responsible for significant healthcare resource utilization (Ihbe-Heffinger et al., 2004; Burke et al., 2011). In a United States–based retrospective cohort study that included 19,139 patients receiving HEC or MEC, 13.7% of patients had a delayed CINV-associated hospital visit and 0.2% had an acute CINV-associated hospital visit (Burke et al., 2011). CINV-associated visits included inpatient (64%), outpatient (26%), and emergency room (<1%) visits.

## Prevention of Delayed Emesis

### NK-1 Receptor Antagonists

The growing understanding of the role of substance P in emesis led to the development of NK-1 receptor antagonists for the treatment of delayed CINV. The first oral NK-1 receptor antagonist, aprepitant, was approved in 2003, followed by fosaprepitant (a prodrug of aprepitant that is administered intravenously), netupitant (administered as a fixed oral combination with the 5-HT_3_ receptor antagonist palonosetron), and rolapitant. The efficacy and tolerability of NK-1 receptor antagonists for prevention of delayed CINV when used in combination with a 5-HT_3_ receptor antagonist and a corticosteroid has been established in a number of randomized controlled trials, as described below. The findings of these trials, along with the demonstrated inability of 5-HT_3_ receptor antagonists to prevent delayed CINV (Aapro, 2005), show the need to incorporate NK-1 receptor antagonists in the treatment of delayed CINV.

The addition of aprepitant to ondansetron plus dexamethasone was shown to increase protection against delayed CINV in patients receiving HEC (Hesketh et al., 2003; Poli-Bigelli et al., 2003) and MEC (Rapoport et al., 2010). In a phase 3, randomized, double-blind study in patients scheduled to receive treatment with high-dose cisplatin, CR rates during the delayed phase were 68% in the aprepitant group and 47% in the standard-therapy group (*p* < 0.001) (Poli-Bigelli et al., 2003). While aprepitant was associated with a significant improvement in the proportion of patients who did not experience delayed vomiting (72 vs. 48%; *p* < 0.01), between-treatment differences in rates of no significant nausea (73 vs. 65%) were not statistically significant. In a similar study, CR rates during the delayed phase were 66% in the aprepitant group and 52% in the standard-therapy group (*p* < 0.001) (Hesketh et al., 2003). As in the previous study, aprepitant had a significant benefit with respect to rates of emesis but not nausea. Benefits on delayed CINV were also reported in patients treated with MEC (including AC regimens) (Rapoport et al., 2010).

Single-dose fosaprepitant was approved for use in delayed CINV based on the results of a phase 3 non-inferiority trial versus aprepitant (administered once daily for 3 days) in patients receiving HEC and treated with background ondansetron and dexamethasone (Grunberg et al., 2011). No significant difference was reported between the fosaprepitant and aprepitant arms with regard to CR rate in the delayed phase (74.3 vs. 76.8%). A recent phase 3 study evaluated the addition of fosaprepitant to ondansetron and dexamethasone in patients receiving non-AC MEC (Weinstein et al., 2016). In this randomized, double-blind, placebo-controlled study, fosaprepitant significantly improved rates of delayed CR (79 vs. 69%; *p* < 0.001) and no emesis (84 vs. 75%; *p* < 0.001). The impact of fosaprepitant on nausea in the delayed phase of this study was not described.

In 2014, netupitant was approved for prevention of CINV. Netupitant is administered as a fixed oral combination with palonosetron (NEPA), and this formulation has been evaluated in phase 3 randomized controlled trials in patients receiving HEC (Hesketh et al., 2014) and AC (considered MEC at the time of the study) (Aapro et al., 2014c). In the HEC population, a CR during the delayed phase was reported in 92% of the NEPA plus dexamethasone group compared with 80% of the control group receiving palonosetron plus dexamethasone (*p* ≤ 0.01), with significant benefits reported in terms of both vomiting and nausea (Hesketh et al., 2014). In the AC study, the percentage of patients with a CR during the delayed phase was significantly higher with NEPA plus dexamethasone than with palonosetron plus dexamethasone (76.9 vs. 69.5%; *p* = 0.0001) (Aapro et al., 2014c). Likewise, NEPA plus dexamethasone was associated with significantly higher rates of no emesis (81.8 vs. 75.6%; *p* = 0.004) and no significant nausea (defined as a reading of <25 mm on a 100-mm horizontal visual analog scale) (76.9 vs. 71.3%; *p* = 0.014).

Rolapitant is the most recent NK-1 receptor antagonist to be approved, and it is licensed for the treatment of delayed CINV associated with initial and repeat courses of emetogenic chemotherapy including, but not limited to, HEC (Varubi, 2015). The efficacy of rolapitant in preventing CINV when added to granisetron plus dexamethasone has been evaluated in two phase 3 clinical trials in patients receiving HEC (Rapoport B.L. et al., 2015) and one phase 3 clinical trial in patients receiving MEC or AC-based chemotherapy (Schwartzberg L.S. et al., 2015). In a pooled analysis of the HEC studies, the addition of rolapitant to active therapy resulted in a 60% improvement in the likelihood of achieving a CR in the delayed phase (71% of rolapitant recipients vs. 60% of active-control recipients; odds ratio 1.6; 95% CI 1.3–2.1; *p* = 0.0001) (Rapoport B.L. et al., 2015). The addition of rolapitant to active therapy also produced a significantly higher rate of no emesis and no clinically significant nausea in the delayed phase.

In the MEC study, rolapitant recipients had a higher rate of CR in the delayed phase than active-control recipients (71 vs. 62%; OR 1.6; 95% CI 1.2–2.0; *p* = 0.0002), and rolapitant was associated with significant benefits in the prevention of vomiting but not of nausea. A prespecified analysis found that the benefit of rolapitant on CR in the delayed phase was maintained irrespective of whether patients were treated with AC. A further analysis in the subgroup of patients treated with carboplatin-based chemotherapy found that the absolute benefit observed with rolapitant (the absolute difference between the proportion of rolapitant and active-control respondents) was 16.7 percentage points for CR in the delayed phase (Hesketh et al., 2016b). Interestingly, in the study mentioned above that showed improved rates of delayed-phase CR and delayed-phase emesis with fosaprepitant in patients receiving non-AC MEC, approximately 53% of these patients were receiving a carboplatin-based chemotherapy regimen (Weinstein et al., 2016). While this study did not stratify efficacy findings by individual MEC agent, it does support the use of NK-1–receptor antagonists in patients receiving carboplatin. This is borne out in the recent update to the MASCC/ESMO guidelines, in which an NK-1 receptor antagonist is recommended in addition to a 5-HT_3_ receptor antagonist and dexamethasone for patients receiving carboplatin (MASCC/ESMO, 2016).

In the trials of rolapitant for the treatment of CINV associated with HEC, more patients receiving rolapitant than active control reported no nausea (≤5 mm on a 100-mm horizontal visual analog scale) in both the overall phase (52 vs. 42%, *p* = 0.0004) and the delayed phase (56 vs. 44%; *p* = 0.0002) (Rapoport B.L. et al., 2015). In one of the trials of aprepitant in patients receiving cisplatin, a greater proportion of patients in the aprepitant group than in the active-control group reported no nausea in the overall phase (49 vs. 39%; *p <* 0.005) and the delayed phase (53 vs. 40%, *p* < 0.05) (Poli-Bigelli et al., 2003), but these effects were not replicated in the second concurrent aprepitant trial in cisplatin-treated patients (Hesketh et al., 2003). Neither rolapitant, aprepitant, nor fosaprepitant significantly increased the number of patients reporting no nausea after treatment with MEC (Rapoport et al., 2010; Schwartzberg L.S. et al., 2015; Weinstein et al., 2016). Trials of NEPA did not include no nausea as an endpoint measure (Aapro et al., 2014c; Hesketh et al., 2014).

Three studies have been published describing the efficacy and safety of aprepitant over multiple cycles of chemotherapy. The first assessed the use of aprepitant over six cycles of cisplatin treatment, using transitional probability models to estimate response rates; it showed that the probability of a CR (no emesis and no significant nausea) was greater in patients receiving aprepitant than active control in the first, fifth, and sixth cisplatin treatment cycles (*p* < 0.05) (de Wit et al., 2003), with drug-related adverse events (AEs) reported in 34% of patients receiving aprepitant versus 25% of those receiving standard therapy. A much larger pooled analysis of the two aforementioned phase 3 trials (Hesketh et al., 2003; Poli-Bigelli et al., 2003), also using transitional probability analyses, found that aprepitant-treated patients were more likely than those receiving standard therapy to exhibit a CR over all six cycles of cisplatin-based therapy (de Wit et al., 2004), with similar rates of drug-related AEs (6 and 4%, respectively). Aprepitant treatment was also associated with a greater probability of CR in each treatment cycle in patients receiving four cycles of MEC (Herrstedt et al., 2005); overall rates of drug-related AEs were not reported.

In a multiple-cycle extension of the phase 3 trial reported by Aapro et al. (2014c), NEPA was associated with superior CR rates compared with palonosetron over four cycles of AC-based chemotherapy (*p* < 0.001 in cycles 2–4), with a similar incidence of AEs observed in each treatment arm (Aapro et al., 2014b). The efficacy and safety of NEPA versus aprepitant over six cycles of chemotherapy in patients receiving MEC or HEC was evaluated in a phase 3 clinical trial (Gralla et al., 2014); overall rates of CR in each cycle were similar for the two treatments (81–91% and 76–88%, respectively), and rates of drug-related AEs were also similar over all cycles (10 and 6%, respectively).

To determine the efficacy and safety of rolapitant over multiple cycles of chemotherapy, a post hoc analysis was carried out on pooled safety and efficacy data from four rolapitant clinical studies (Rapoport et al., 2016): the phase 2 dose-determining study of rolapitant in patients receiving HEC (Rapoport B. et al., 2015) and the three previously mentioned phase 3 trials (Rapoport B.L. et al., 2015; Schwartzberg L.S. et al., 2015). Rates of emesis were lower in the pooled population of patients receiving rolapitant than in those receiving placebo in all chemotherapy cycles after the first (cycles 2–6), and a higher proportion of patients in the pooled rolapitant group reported no nausea interfering with daily life and the combined measure of no emesis or interfering nausea over cycles 2–5 (Rapoport et al., 2016). The incidence of treatment-emergent AEs was low and was similar in both groups after cycle 1 (rolapitant, 5.5%; control, 6.8%), and it did not increase with each subsequent cycle.

NK-1 receptor antagonists are generally well tolerated; the most commonly reported treatment-emergent AEs with NK-1 receptor antagonists in clinical trials included headache, constipation, fatigue, and hiccups, which appeared with a similar frequency as in active-control groups (Navari, 2016).

Differences in pharmacokinetic properties between NK-1 receptor antagonists may affect their dosing (**Table 2**). Aprepitant has a relatively short half-life of 9–13 h, requiring daily dosing across days 1–3 of each cycle (Emend, 2015), whereas the half-lives for NEPA and rolapitant are approximately 80 and 180 h, respectively, and each agent is administered as a single dose 1–2 h prior to chemotherapy (Akynzeo, 2015; Varubi, 2015). All of the NK-1 receptor antagonists, with the exception of rolapitant, inhibit or induce CYP3A4. A reduced dose of dexamethasone (a CYP3A4 substrate) should be administered with aprepitant and NEPA, but it is not required with rolapitant. Rolapitant does not inhibit or induce CYP3A4, with no effect shown on the pharmacokinetics of the sensitive CYP3A4 substrate midazolam (Poma et al., 2013). Rolapitant is a moderate inhibitor of CYP2D6 and an inhibitor of breast cancer resistance protein (BCRP) and P-glycoprotein, and its concomitant use with substrates of these enzymes that have a narrow therapeutic index should be avoided. However, in an integrated safety analysis of randomized trials, the incidence of treatment-emergent AEs was similar in the rolapitant and control arms in patients who used concomitant CYP2D6, BCRP, or CYP3A4 substrate drugs (Barbour et al., 2015).

**Table 2:** Recommended dosing of NK-1 receptor antagonists.

NK-1 receptor antagonist	Day 1	Days 2–3
Aprepitant	Single oral 125-mg dose prior to chemotherapy	Oral dose of 80 mg once daily on days 2 and 3
Fosaprepitant	Single intravenous 150-mg dose prior to chemotherapy	–
Netupitant	Single oral 300 mg netupitant/0.5-mg palonosetron dose prior to chemotherapy	–
Rolapitant	Single oral 180-mg dose prior to chemotherapy	–

### Other Antiemetics for Delayed Emesis

Corticosteroids have been used as prophylaxis against CINV, particularly delayed CINV, for many years, although their exact mechanism of action is unknown. The antiemetic efficacy of 5-HT_3_ receptor antagonists (or dopamine antagonists) increases when they are used in combination with corticosteroids (Grunberg, 2007); therefore, these agents are typically administered concurrently.

Olanzapine, an atypical antipsychotic drug that blocks dopaminergic, serotonergic, adrenergic, and histamine receptors, has been evaluated in combination with 5-HT_3_ receptor antagonist and corticosteroid for delayed CINV prophylaxis (Tan et al., 2009; Navari et al., 2011). Benefits with this agent have been reported for both acute and delayed nausea control (Abe et al., 2016; Chiu et al., 2016; Navari et al., 2016). The National Comprehensive Cancer Network (2016) guidelines include olanzapine with a 5-HT_3_ antagonist and corticosteroid as a treatment option for prevention of both HEC- and MEC-associated CINV. The recommendations also include consideration of replacing NK-1 receptor antagonist–containing regimens with an olanzapine-containing regimen for management of breakthrough emesis.

## Current CINV Prophylaxis Guidelines

Several evidence-based guidelines for the prevention of CINV have been developed by international professional societies (Hesketh et al., 2016b; MASCC/ESMO, 2016; National Comprehensive Cancer Network, 2016), which are relatively consistent in their key recommendations (summarized in **Table 3**). In general, the guidelines recommend prescribing a NK-1 receptor antagonist along with a 5-HT_3_ receptor antagonist and dexamethasone for prevention of CINV in patients receiving HEC, and a 5-HT_3_ receptor antagonist and dexamethasone in patients receiving MEC (Hesketh et al., 2016b; MASCC/ESMO, 2016; National Comprehensive Cancer Network, 2016). The National Comprehensive Cancer Network and the American Society of Clinical Oncology also recommend that an NK-1 receptor antagonist be considered for patients treated with MEC, particularly those with additional risk factors for CINV (Hesketh et al., 2016b; National Comprehensive Cancer Network, 2016). The authors of these guidelines have made a concerted effort to define antiemetic regimens that cover both the acute and delayed phases of CINV.

**Table 3:** Summary of evidence-based guidelines for chemotherapy-induced nausea and vomiting (CINV) prophylaxis with intravenous chemotherapy.

Emetic risk category	Guideline recommendation
High (including AC combinations)	NK-1 receptor antagonist + 5-HT_3_ receptor antagonist + dexamethasone (Hesketh et al., 2016b; MASCC/ESMO, 2016; National Comprehensive Cancer Network, 2016) *or* Olanzapine + 5-HT_3_ receptor antagonist + dexamethasone (National Comprehensive Cancer Network, 2016)
Moderate	5-HT_3_ receptor antagonist + dexamethasone (± NK-1 receptor antagonist^a^) (Basch et al., 2011; National Comprehensive Cancer Network, 2016) *or* Olanzapine + 5-HT_3_ receptor antagonist + dexamethasone (National Comprehensive Cancer Network, 2016) *or* 5-HT_3_ receptor antagonist + dexamethasone (MASCC/ESMO, 2016)
Low	Dexamethasone (Basch et al., 2011; MASCC/ESMO, 2016; National Comprehensive Cancer Network, 2016) *or* Dopamine receptor antagonist OR 5-HT_3_ receptor antagonist (MASCC/ESMO, 2016; National Comprehensive Cancer Network, 2016)
Minimal	No prophylactic antiemetic (Basch et al., 2011; MASCC/ESMO, 2016; National Comprehensive Cancer Network, 2016)

^a^*An NK-1 receptor antagonist should be added for patients with additional risk factors or who are failing 5-HT_3_ receptor antagonist + dexamethasone (National Comprehensive Cancer Network, 2016). The NK-1 receptor antagonist recommended in the ASCO guidelines is aprepitant (Basch et al., 2011). 5-HT_3_, 5-hydroxytryptamine type 3; AC, anthracycline–cyclophosphamide; CINV, chemotherapy-induced nausea and vomiting; NK-1, neurokinin-1*.

Adherence to antiemetic guidelines improves the control of acute and delayed CINV (Aapro et al., 2012); however, such adherence is suboptimal across a range of settings (Aapro et al., 2012; Burmeister et al., 2012; Gomez et al., 2013; Gilmore et al., 2014; Jordan et al., 2014; Yu et al., 2015). Nonadherence to guidelines may include the failure to use NK-1 receptor antagonists as part of the antiemetic regimen (Gomez et al., 2013; Gilmore et al., 2014) and the overuse of 5-HT_3_ receptor antagonists for prevention of delayed CINV (Burmeister et al., 2012).

## Conclusion and Future Directions

At present, antiemetic therapy recommendations are based largely on the emetogenic potential of the chemotherapy regimen, with less consideration of individual risk factors. Incorporation of personal risk factors may allow better prediction of CINV and improve personalized management of CINV. Indices that can discriminate between patients at high and low risk of both acute and delayed CINV are currently in development (Dranitsaris et al., 2013) and are being validated in randomized controlled trials. For example, patients with early-stage breast cancer receiving AC were randomized to risk-model guided (RMG) antiemetic prophylaxis or physician’s choice of therapy. Benefits were seen in both the acute and delayed phase with RMG therapy: specifically, significantly more patients in the RMG group than the physician’s choice group reported no delayed nausea (39.6 vs. 30.7%; *p* = 0.01) and no delayed vomiting (87.1 vs. 78.0%; *p* < 0.001) (Clemons et al., 2016). The development of algorithms with high sensitivity and specificity to aid clinical decision making may improve CINV prophylaxis, particularly in the delayed phase.

There are several possible explanations for the persistence of delayed CINV even in the absence of acute CINV. The delayed phase may be inherently resistant to treatment, appropriate prophylactic antiemetics may be inadequately prescribed because of underestimation of delayed CINV control, or patients may be nonadherent to prescribing instructions when pills need to be taken at home. Whatever the case, delayed CINV continues to be a treatment challenge. Effective treatment of nausea over both the acute and delayed phases also remains an unmet clinical need in both patients receiving HEC and those receiving MEC (Ng et al., 2015), although the addition of olanzapine to standard triple therapy of an NK-1 receptor antagonist, a 5-HT_3_ receptor antagonist, and dexamethasone has shown benefit in patients receiving cisplatin- or cyclophosphamide-doxorubicin–based HEC (Abe et al., 2016; Chiu et al., 2016; Navari et al., 2016). Identifying patients at risk of delayed CINV and initiating prophylaxis with triple therapy before administration of chemotherapy is likely to improve clinical outcomes and patients’ daily lives.

## Author Contributions

BLR developed the concept of this work, critically revised all drafts, gave final approval for submission of the final version for publication, and is accountable for all aspects of the work.

## Conflict of Interest Statement

BLR has received honoraria and expenses from Herron, Merck and Co. and Tesaro, has sat on advisory boards for Herron, Merck and Co. and Tesaro and has received research funding from Merck and Co. and Tesaro.
